# A retrospective study of predictive factors for unexpectedly prolonged or shortened progression-free survival and overall survival among patients with metastatic renal cell carcinoma who received first-line targeted therapy

**DOI:** 10.1186/s12885-016-2615-4

**Published:** 2016-08-02

**Authors:** Sung Han Kim, Sohee Kim, Jungnam Joo, Ho Kyung Seo, Jae Young Joung, Kang Hyun Lee, Jinsoo Chung

**Affiliations:** 1 Department of Urology, Center for Prostate Cancer, Hospital of National Cancer Center National Cancer Center, Goyang, Korea; 2 Biometric Research Branch, Clinical Research Coordination Center, Hospital of National Cancer Center National Cancer Center, Goyang, Korea; 3 Center for Prostate Cancer, National Cancer Center, 323 Ilsan-ro, Ilsandong-gu, Goyang-si, Gyeonggi-do 410-769 Republic of Korea

**Keywords:** Renal cell carcinoma, Neoplasm metastasis, Prognosis, Overall survival, Progression free survival, Molecular targeted therapy

## Abstract

**Background:**

To identify predictors of prolonged or shortened progression-free survival (PFS) and overall survival (OS) among patients with metastatic renal cell carcinoma (mRCC) who received first-line targeted therapies.

**Methods:**

This retrospective study included 146 patients with mRCC who were treated during 2007–2015. These patients were divided into a group with the worst response (WG), an expected group (EG), and a group with the best response (BG), based on their PFS (≤3 monthsnths, 3–18 monthsnths, and >18 monthsnths, respectively) and OS (<1 year, 1–3 years, and >3 years, respectively). To identify significant predictive factors, the BG and WG were compared to the EG using the Memorial Sloan Kettering Cancer Center and Heng risk models.

**Results:**

The overall PFS and OS were 9.3 months and 16.4 months, respectively. The median PFS for the WG (41.8 %), EG (45.9 %), and BG (12.3 %) were 2.7 months, 9.3 months, and 56.6 months, respectively, and the median OS for the WG (45.9 %), EG (35.6 %), and BG (18.5 %) were 5.5 months, 21.6 months, and 63.1 months, respectively; these outcomes were significantly different (*p <* 0.001). Nephrectomy (odds ratio [OR]: 7.15) was a significant predictor of PFS in the BG, and the significant predictors of OS in the BG were MSKCC intermediate risk (OR: 0.12), poor risk (OR: 0.04), and a disease-free interval of <1 year (OR: 0.23) (all, *p <* 0.05). Anemia (OR: 3.25) was a significant predictor of PFS in the WG, and the significant predictors of OS were age (OR: 1.05), anemia (OR: 4.13), lymphocytopenia (OR: 4.76), disease-free interval of <1 year (OR: 4.8), and synchronous metastasis (OR: 3.52) (all, *p <* 0.05).

**Conclusion:**

We identified several significant predictors of unexpectedly good and poor response to first-line targeted therapy among patients with mRCC.

**Electronic supplementary material:**

The online version of this article (doi:10.1186/s12885-016-2615-4) contains supplementary material, which is available to authorized users.

## Background

Research regarding the molecular biology of renal cell carcinoma (RCC) and the subsequent introduction of targeted therapeutic agents (TTs) have resulted in improved treatment guidelines for metastatic RCC (mRCC), and significantly improved progression-free survival (PFS) and overall survival (OS) [1, 2]. However, the appropriate treatment for mRCC in each case remains unclear, as the tumor’s heterogeneity can affect the clinical outcomes after TT treatment, and it is difficult to accurately predict individual patients’ prognoses. Therefore, it remains challenging to optimize therapeutic outcomes using personalized therapy.

Diverse criteria are used to stratify patients’ prognoses, evaluate therapeutic responses, and determine patients’ eligibility for TTs, and these criteria are used to help predict the patients’ PFS and OS after TT treatment [3, 4]. Among the various evaluation tools and prognostic models, the RECIST criteria [5] are the best known and most commonly used evaluation tools for radiologically stratifying patients with solid tumors who received TT treatment, based on the responses of their primary tumor and metastatic lesions [4, 6]. Furthermore, the Memorial Sloan Kettering Cancer Center (MSKCC) [7, 8] and the International Metastatic Renal Cell Carcinoma Database Consortium (IMDC, also named as Heng) risk criteria [9] have been used in clinical prognostic models that predict the response to TT among patients with mRCC. However, even with these tools, clinicians may encounter difficulties in identifying patients who might experience clinical outcomes that significantly deviate from the expected outcomes. Therefore, the present study aimed to evaluate the clinicopathological characteristics of patients with mRCC who experience unexpectedly prolonged or shortened PFS and OS, and to identify significant predictors of unexpected clinical responses to first-line TTs.

## Methods

This retrospective study was approved by the institutional review board of the Research Institute and Hospital National Cancer Center (approval no. NCC2014-0155), and the requirement for informed consent was waived. All patient data were anonymized and de-identified prior to our analysis. All study protocols were performed in accordance with the ethical tenets of the Declaration of Helsinki.

We identified 146 patients with mRCC and an intact contralateral kidney, who were treated using first-line TTs without any prior systemic treatment between January 2007 and April 2015. All included patients had complete follow-up and medical history data, and none of the patients discontinued their first-line TT due to Grade 3 or higher adverse events. The specific first-line TT was selected at the discretion of the treating urologist (JC), who considered each patient’s histopathology, disease status, medical condition, and the wishes of the patient and their family after a comprehensive discussion regarding the anticipatory efficacy and adverse events of each TT. Each cycle of sunitinib consisted of a daily 50-mg oral dose over a 4-week period, which was followed by a 2-week hiatus. Each cycle of sorafenib consisted of twice-daily 400-mg oral doses for a 6-week period. Each cycle of pazopanib consisted of a daily 800-mg oral dose over a 6-week period. Each cycle of temsirolimus consisted of a weekly 25-mg intravenous infusion over a 6-week period. All patients underwent a complete evaluation after every two cycles of TT, which included a total physical evaluation, blood tests, and radiological examinations. The radiological examinations included contrast-enhanced computed tomography and/or positron emission tomography–computed tomography and bone scans to evaluate treatment response, which was based on the RECIST criteria (version 1.1) [5]. Treatment was continued until disease progression was identified.

The 146 patients were grouped according to their PFS and OS, and the cut-offs were selected based on previously published representative findings that included a PFS of 4–18.8 months and an OS of 11.9–33.1 months [1, 2, 10–12]. Therefore, to stratify patients as having experienced unexpectedly prolonged or shortened OS and PFS, we categorized the patients using PFS cut-offs of 3 months and 18 months, and OS cut-offs of 1 year and 3 years. The upper PFS cut-off value was not set to 17 months, as none of the patients exhibited a PFS of approximately 17 months during their first-line TT treatment. Thus, the patients were grouped according to whether they had experienced the worst survival outcomes (WG; PFS: ≤3 months, OS: <1 year), the normally expected outcomes (EG; PFS: 3–18 months, OS: 1–3 years), or the best survival outcomes (BG; PFS: >18 months, OS: >3 years).

Differences and associations between the baseline characteristics were examined using the chi-square test, Fisher’s exact test, and the Kruskal-Wallis test, as appropriate. Binary logistic regression models were used to calculate the odds ratios (ORs) and 95 % confidence intervals (CIs) for the factors that significantly affected the BG and WG outcomes, compared to the EG outcomes. Only factors with a *p*-value of <0.10 in the univariable analysis were subsequently evaluated in the multiple logistic regression analysis, using backwards stepwise selection with a significance level of 0.10. Variables with large amounts of missing data (>20 % of patients) were excluded from the multivariable analysis (clinical T and N stages, and pathological T, N, and M stages). The times to progression and death were evaluated using Kaplan-Meier curves and the log-rank test. All analyses were performed using Stata software (version 13.1; Stata Corp., College Station, TX, USA), and differences with a *p*-value of <0.05 were considered statistically significant.

## Results

**Fig. 1:**
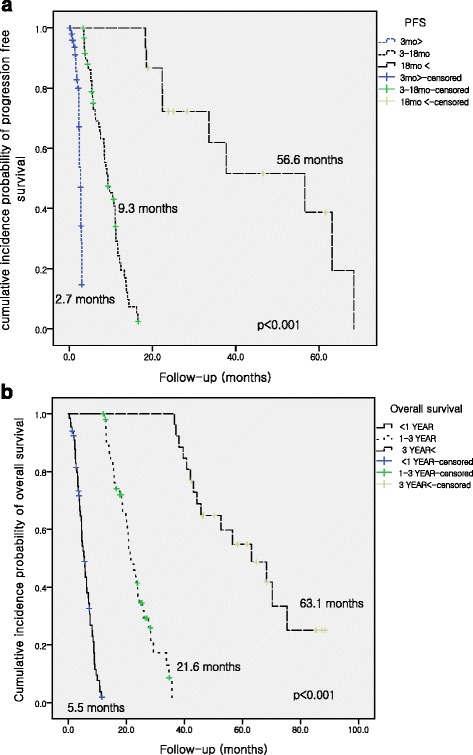
The Kaplan-Meier curves for (**a**) progression-free survival (PFS) and (**b**) overall survival (OS) among the control group and the groups with the worst and best responses to first-line targeted therapy

The disease control rate, objective response rate, PFS, and OS among all 146 patients were 70.6 %, 46.3 %, 9.3 months (95 % CI: 7.3–11.2 months), and 16.4 months (95 % CI: 12.2–20.8 months), respectively. Seven patients (6.4 %) achieved complete response, 15 patients (10.3 %) were still being treated with first-line TT (i.e., stable disease or partial response), and 105 patients (71.9 %) exhibited a progression-free interval of <1 year. The baseline characteristics of the patients in the WG, EG, and BG are summarized in Tables 1 and 2. The median PFS for the WG (*n =* 61, 41.8 %), EG (*n =* 67, 45.9 %), and BG (*n =* 18, 12.3 %) were 2.7 months (95 % CI: 2.4–2.9 months), 9.3 months (95 % CI: 8.3–11.1 months), and 56.6 months (95 % CI: 22.4–68.4 months), respectively (Fig. 1a). The median OS of the WG (*n =* 65, 45.9 %), EG (*n =* 52, 35.6 %), and BG (*n =* 27, 18.5 %) were 5.5 months (95 % CI: 4.5–6.9 months), 21.6 months (95 % CI: 19.8–24.4 months), and 63.1 months (95 % CI: 44.3–75.4), respectively (Fig. 1b). These survival outcomes were significantly different (all, *p <* 0.001).

**Table 1:** Clinicopathological characteristics of the worst group (*n =* 61, 41.8 %), expected group (*n =* 67, 45.9 %), and the best group (*n =* 18, 12.3 %), according to their progression-free survival

Variables (N, %)	Worst Group (≤3 mo)	Control Group (>3 and ≤18 mo)	Best Group (>18 mo)	*p*-value
Age	58.5 ± 10.9	58.0 ± 11.2	60.5 ± 11.1	0.697
Gender Male/Female	45/1 (73.8/26.2)	55/12 (82.1/37.7)	17/1 (94.4/5.6)	0.140
Body mass index (kg/m^2^)	23.4 ± 3.1	23.2 ± 2.7	23.9 ± 2.0	0.647
MSKCC criteria				<0.001
Favorable	3 (5.9)	5 (9.3)	6 (37.5)	
Intermediate	30 (58.8)	42(77.8)	10 (62.5)	
Poor	18 (35.3)	7 (13.0)	0	
Heng criteria				0.003
Favorable	5 (8.9)	9 (15.8)	8 (47.1)	
Intermediate	38 (67.9)	42 (73.7)	9 (52.9)	
Poor	13 (23.2)	6 (10.5)	0	
ECOG 0	54 (93.1)	59 (100)	18 (100)	0.078
1	4 (6.9)	0	0	
Metastatic site				
Lung	48 (80.0)	57 (89.1)	14 (77.8)	0.268
Liver	15 (25.0)	9 (14.8)	1 (5.9)	0.139
Lymph node	32 (53.3)	31 (49.2)	9 (50.0)	0.918
Bone	19 (32.2)	21 (34.4)	6 (35.3)	0.968
Brain	7 (11.7)	7 (11.7)	2 (12.5)	1.000
Other metastasis	13 (22.0)	12 (19.7)	2 (11.8)	0.691
Nephrectomy	28 (45.9)	35 (52.2)	16 (88.9)	0.004
Embolization	3 (4.9)	3 (4.5)	2 (11.1)	0.455
Clinical T stage				0.371
T1	7 (16.3)	5 (10.7)	2(16.7)	
T2	4 (9.3)	11(23.4)	0	
T3	22 (51.1)	15 (32.0)	3 (25.0)	
T4	3 (7.0)	6(12.8)	3 (25.0)	
Tx	7(16.3)	10 (21.3)	4 (33.3)	
N1	9 (18.8)	9 (19.1)	4 (28.6)	0.570
synchronous metastasis	35 (59.3)	50 (75.8)	12 (66.7)	0.144
Fuhrman nuclear grade				0.767
1–2	14 (34.1)	8 (36.7)	3 (25.0)	
3–5	27 (65.9)	31 (63.3)	9 (75.0)	
Histology				0.701
Clear cell type	45 (77.6)	55(87.3)	11 (73.3)	
Non-clear cell type	2(3.4)	1 (16)	0	
Chromophobe with clear cell	2 (3.3)	3 (4.5)	1 (5.6)	
Papillary with clear cell	7 (12.2)	2 (3.5)	2 (14.4)	
unknown type	2 (3.4)	2 (3.2)	1(6.7)	
Sarcomatoid presence	5(8.8)	4 (6.5)	1 (6.7)	0.895
Treatment				0.877
Sunitinib	43 (70.5)	45 (67.2)	13 (72.2)	
Sorafenib	8(13.1)	8 (11.9)	1 (5.6)	
Pazopanib	8 (13.1)	13(19.4)	4 (22.2)	
Temsirolimus	2 (3.3)	1 (1.5)	0	
RECIST response				<0.001
CR	0	2 (3.3)	5 (29.4)	
PR	2 (6.5)	27 (44.3)	8 (47.1)	
SD	7 (22.6)	23 (37.7)	3 (17.6)	
PD	22 (71.0)	19 (14.8)	1 (5.9)	
Laboratory findings				
Leukocytosis/Leucopenia	15/0 (26.3/0)	8/3(13.8/5.2)	2/1(11.8/5.9)	0.140
Anemia	43 (75.4)	28 (48.3)	5 (29.4)	<0.001
Thrombocytosis/penia	11/2(19.3/3.5)	7/2 (12.1/3.4)	0/0	0.279
Neutrophilia/penia	14/0 (24.6/0)	7/1(12.1/1.7)	1/1 (5.9/5.9)	0.089
Lymphocytosis/penia	2/27 (3.5/47.4)	5/14 (8.6/24.1)	1/1 (5.9/5.9)	0.004
Hyper/hypocalcemia	3/11 (5.3/19.3)	3/3 (5.2/5.2)	0/0	0.059
Hypoalbuminemia	12 (20.3)	0	0	<0.001
LDH elevated	8 (14.0)	4 (6.9)	0	0.190
Neutrophil percent high/low	113 (19.3/5.3)	5/7 (8.6/12.1)	1/5 (5.9/29.4)	0.046
Progression-free survival (mo.)	2.7 (0.1–3.0)	9.3 (3.3–16.5)	56.6 (18.3–68.4)	<0.001
Overall survival (mo.)	6.9 (0.3–58.4)	18.6 (4.0–70.3)	68.3 (18.3–78.4)	<0.001

**Table 2:** Clinicopathological characteristics of the worst group (*n =* 67, 45.9 %), expected group (*n =* 52 35.6 %), and best group (*n =* 27, 18.5 %), according to their overall survival

Variables (N, %)	Worst Group (<1 y)	Control Group (1–3 y)	Best Group (>3 y)	p-value
Age (years)	60.5 ± 10.7	57.1 ± 11.5	56.3 ± 10.3	0.136
Gender (Male/Female)	50/17 (74.6/25.4)	45/7 (86.5/13.5)	22/5 (81.5/18.5)	0.266
Body mass index (kg/m^2^)	22.9 ± 2.8	23.7 ± 2.5	24.3 ± 2.9	0.096
MSKCC criteria	59 (88.1)	43 (100)	19 (73.1)	<0.001
Favorable risk	0	4 (9.3)	10 (52.6)	
Intermediate risk	39 (66.1)	34 (79.1)	9 (47.4)	
Poor risk	20(33.9)	5 (11.6)	0	
Heng criteria	64 (95.5)	44 (84.6)	22 (81.5)	<0.001
Favorable risk	2 (3.1)	9(20.5)	11 (50.0)	
Intermediate risk	46 (71.9)	32 (72.7)	11 (50.0)	
Poor risk	16 (25.0)	3 (6.8)	0	
ECOG 0	61(93.8)	47 (100)	23(100)	0.109
1	4 (6.2)	0	0	
Metastatic site				
Lung	54 (81.8)	42(84.0	23 (88.5)	0.738
Liver	18(27.3)	6 (12.2)	1 (4.3)	0.020
Lymph node	33 (49.3)	17 (36.2)	8 (33.3)	0.064
Bone	23 (34.8)	16 (34.0)	6 (25.0)	0.612
Brain	9 (13.6)	5 (10.6)	2 (8.3)	0.758
Other metastasis	18 (27.3)	5 (10.6)	4 (16.7)	0.083
Nephrectomy Embolization	23(34.3) 4 (5.9)	32 (61.5) 1 (1.9)	24 (88.9) 3(11.1)	<0.0010.146
Clinical T stage	43 (64.2)	35 (67.3)		0.179
T1	4 (10.8)	6 (17.2)	3 (18.8)	
T2	3 (8.1)	8(20.0)	7 (12.5)	
T3	15 (40.5)	11 (31.5)	9 (31.3)	
T4	8 (21.6)	3 (8.6)	0 (18.8)	
Tx	7 (18.9)	7 (20.0)	2(18.8)	
N1	13 (31.0)	6 (14.6)	5 (29.4)	0.229
Synchronous metastasis	58(87.9)	31 (60.8)	8 (30.8)	0.001
Fuhrman nuclear grade	41 (61.2)	39 (75.0)	22 (81.5)	0.460
1–2	13 (31.7)	12 (30.8)	10 (45.5)	
3–5	28 (68.3)	27 (69.2)	12 (54.5)	
Histology	63 (94.0)	50 (96.2)	26 (96.3)	0.581
Clear cell type	50 (79.4)	44 (89.8)	17 (70.8)	
Non-clear cell type	2 (3.2)	1 (2.0)	0	
Chromophobe with clear cell	2 (3.0)	2 (3.8)	2 (7.4)	
Papillary with clear cell	7 (6.5)	1 (2.3)	3 (5.1)	
Unknown type	2 (3.2)	1 (2.0)	2 (8.3)	
Sarcomatoid presence	3 (4.8	1 (2.0)	2(8.3)	0.168
Treatment				0.430
Sunitinib	42 (62.7)	37 (71.2)	22(81.5)	
Sorafenib	9 (13.4)	6 (11.5)	2 (7.4)	
Pazopanib	13 (19.4)	9 (17.3)	3 (11.1)	
Temsirolimus	3 (4.5)	0	0	
RECIST response				<0.001
CR	0	4 (9.1)	3 (15.0)	
PR	9 (20.0)	19 (43.2)	9 (45.0)	
SD	11 (24.4)	14 (31.8)	8 (40.0)	
PD	25 (55.6)	7 (15.9)	0	
Laboratory findings				
Leukocytosis/Leucopenia	19/1 (28.4/1.6)	4/2 (8.9/4.4)	2/1(7.4/3.7)	0.030
Anemia	51 (76.4)	17 (37.8)	8 (29.6)	<0.001
Thrombocytosis/penia	14/2(20.9/3.0)	4/2 (8.9/4.4)	0/0	0.041
Neutrophilia/penia	18/0 (26.9/0)	2/1 (4.4/2.2)	2/1(7.4/3.7)	0.002
Lymphocytosis/penia	1/37 (1.5/55.2)	4/4 (8.9/8.9)	3/1 (11.1/3.7)	<0.001
Hyper/hypocalcemia	5/11 (7.4/16.4)	1/2 (2.2/4.4)	0/1 (0/3.7)	0.077
Hypoalbuminemia	12(17.9)	0	0	0.002
LDH elevated	10 (14.9)	2(4.4)	0	0.051
Neutrophil percent high/low	16/1 (23.9/1.5)	0/8(0/17.8)	1/6 (3.7/22.2)	<0.001
PFS (mo.)	2.7 (1–9.3)	9.5 (1–28.3)	12.2 (1–68.4)	<0.001
OS (mo.)	5.5 (0.3–11.6)	21.6 (12.1–35.7)	63.1 (36.6–88.4)	<0.001

The correlation and parametric trend tests for PFS and OS revealed that each group’s PFS and OS were significantly correlated (Pearson’ correlation coefficient: 0.6283, and non-parametric trend test, *p <* 0.001). The correlation percentages for the BG, EG, and WG were 50 % (*n =* 9, PFS: >18 months, OS: >3 years), 49.3 % (*n =* 33, PFS: 3–18 months, OS: 1–3 years), and 72.1 % (*n =* 44, PFS: ≤3 months, OS: <1 year).

When we compared the BG and EG using the complete MSKCC risk evaluation, only nephrectomy (OR: 7.15, 95 % CI: 1.43–35.67) was a significant predictor of PFS in the multivariate analysis (*p =* 0.016) (Table 3, see also Additional file 1: Table S1). The significant predictors of OS were MSKCC intermediate risk (OR: 0.12, 95 % CI: 0.003–0.049), MSKCC poor risk (OR: 0.04, 95 % CI: 0.01–0.87), and a disease-free interval of <1 year (Heng, OR: 0.23, 95 % CI: 0.07–0.73) (all, *p <* 0.05) (Table 4, see also Additional file 1: Table S2).

**Table 3:** Predictive factors for progression-free survival after comparing the expected group and the group with the best response to therapy

	Univariate			Multivariate					
				MSKCC risk patients			Heng risk patients		
Variables	OR	*P*-value	95 % CI	HR	*P*-value	95 % CI	HR	*P*-value	95 % CI
Heng Intermediate risk group	0.25	0.019	0.08–0.8				0.32	0.083	0.09–1.16
Poor	0.09	0.111	0.01–1.76				0.25	0.414	0.01–7.0
Nx	7.31	0.012	1.56–34.33	7.15	0.016	1.43–35.67	3.90	0.076	0.87–17.56

**Table 4:** Predictive factors for overall survival after comparing the expected group and the group with the best response to therapy

	Univariate			Multivariate					
				MSKCC risk patients			Heng risk patients		
Variables	OR	*P*-value	95 % CI	HR	*P*-value	95 % CI	HR	*P*-value	95 % CI
MSKCC Intermediate	0.12	0.001	0.03–0.44	0.12	0.003	0.03–0.49			
Poor	0.04	0.040	0.01–0.86	0.04	0.041	0.01–0.87			
DFI < 1 year	0.22	0.003	0.08–176.29				0.23	0.013	0.07–0.73

When we compared the WG (*n =* 105) and EG (*n =* 113), the only significant predictor of PFS was anemia (MSKCC, OR: 3.25, 95 % CI: 1.41–7.52; Heng, OR: 2.87, 95 % CI: 1.23–6.66; both, *p <* 0.05) (Table 5, see also Additional file 1: Table S3). The significant predictors of OS were age (MSKCC, OR: 1.05, 95 % CI: 1.01–1.1), anemia (MSKCC, OR: 4.13, 95 % CI: 1.44–11.8; Heng, OR: 4.61, 95 % CI: 1.68–12.66), lymphocytopenia (MSKCC, OR: 4.76, 95 % CI: 1.25–18.17; Heng, OR: 5.26, 95 % CI: 1.44–19.14), a disease-free interval of <1 year (MSKCC, OR: 4.8, 95 % CI: 1.1–20.9), and synchronous metastasis (MSKCC, OR: 3.52, 95 % CI: 1.07–11.61) (all, *p <* 0.05) (Table 6, see also Additional file 1: Table S4).

**Table 5:** Predictive factors for progression-free survival after comparing the expected group and the group with the worst response to therapy

	Univariate			Multivariate					
				MSKCC risk patients			Heng risk patients		
Variables	OR	*P*-value	95 % CI	HR	*P*-value	95 % CI	HR	*P*-value	95 % CI
Hemoglobin low	3.29	0.003	1.49–7.27	3.25	0.006	1.41–7.52	2.87	0.014	1.23–6.66
Platelet high	1.75	0.288	0.62–4.91						
low	1.11	0.916	0.15–8.24						
Lymphocyte high	0.56	0.503	0.1–3.08				0.26	0.242	0.03–2.47
Low	2.69	0.016	1.2–6.02				2.05	0.098	0.88–4.78

**Table 6:** Predictive factors for overall survival after comparing the expected group and the group with the worst response to therapy

	Univariate			Multivariate					
				MSKCC risk patients			Heng risk patients		
Variables	OR	*P*-value	95 % CI	HR	*P*-value	95 % CI	HR	*P*-value	95 % CI
Age	1.03	0.099	0.99–1.06	1.05	0.045	1.01–1.10			
Hemoglobin low	6.46	0.000	2.74–15.22	4.13	0.008	1.44–11.8	4.61	0.003	1.68–12.66
Lymphocyte high	0.36	0.368	0.04–15.22	0.22	0.215	0.02–2.42	0.18	0.160	0.02–1.95
Low	13.16	0.000	4.18–9.34	4.76	0.022	1.25–18.17	5.26	0.012	1.44–19.14
DFI < 1 year	3.81	0.007	1.43–10.15	4.80	0.036	1.10–20.9			
synchronous metastasis	4.68	0.001	1.85–11.84	3.52	0.039	1.07–11.61	3.17	0.057	0.97–10.41

## Discussion

The shift to TTs for treating mRCC has greatly improved the PFS of patients with mRCC. However, TTs are rarely curative and therapeutic resistance develops after 6–11 months of first-line TT treatment, which eventually leads to disease progression within 4–18.8 months; thus, only a few studies have reported significant improvements in OS [1, 2, 10, 11]. However, the absence of any significant improvements in OS are mainly related to the confounding effects of crossover to active treatment from the placebo/comparator arm. [13] Nevertheless, TT resistance and disease control are addressed via sequential therapy using various combinations of TTs, which provide a general OS of 11.9–33.1 months, and an OS of 9.0–10.9 months for patients with poor-risk features [9, 10, 12, 14, 15].

In the present study, we used PFS cut-off values of 3 months and 18 months, and OS cut-off values of 1 year and 3 years, in order to identify the patients that experienced unexpectedly prolonged or shortened survival outcomes [1, 2, 10, 12]. The cut-off for unexpectedly prolonged PFS was selected based on a review of sorafenib and sunitinib by Porta et al. [13], and a study by Buchler et al. that reported a PFS of 17.7 months among patients who received sunitinib followed by sorafenib (*n =* 138), and 18.8 months among patients who received sorafenib followed by sunitinib (*n =* 122) [16]. Another review article [12] reported that a study of sorafenib from the Nexavar Charity Patient Aid Program provided a PFS of 17.6 months with a 95 % disease control rate. The OS cut-off was supported by data from the SWITCH study, which reported an OS of 31.5 months for the sorafenib-sunitinib group and an OS of 30.2 months for the sunitinib-sorafenib group [17]. Furthermore, Tomita et al. reported that their first-line TT group (*n =* 25, a median of six 6-week cycles) achieved an OS of 33.1 months, and their pretreated group (*n =* 26; 9.5 cycles of TT) achieved an OS of 32.5 months [12]. Therefore, we compared the correlations between PFS and OS in each group, and found that these outcomes were well correlated. Interestingly, the WG exhibited the greatest correlation between PFS and OS (72.1 % of patients), while the BG and EG only exhibited correlations for 50 % of their patients.

In the present study, the overall disease control rate (70.6 %), objective response rate (46.3 %), and median PFS (9.3 months, 95 % CI: 7.3–11.2 months) were similar to those of other previously published series (69–79 %, 24–32 %, and PFS: 5.5–11.1 months for first-line sunitinib [11, 18], sorafenib [11, 19, 20], and pazopanib [21], respectively). In contrast, the median OS (16.4 months, 95 % CI: 12.2–20.8 months) was shorter than those in previous TT trials (22.9–26.4 months) [10, 12, 13]. This discrepancy may be related to the fact that the previous studies generally included patients who had undergone nephrectomy and exhibited clear cell histology, while the present study included relatively small proportions of patients who had undergone nephrectomy (54.1 %), exhibited sarcomatoid histology (6.8 %), exhibited non-clear cell histology (18.4 %), or had poor- or unknown-risk features (30.0–34.2 %) according to the MSKCC and Heng criteria.

Our multivariate analyses revealed that nephrectomy (MSKCC, HR: 7.15) was the only significant predictor of PFS in the BG, and that anemia (MSKCC, HR: 3.25; Heng, HR: 2.87) was the only significant predictor of PFS in the WG (all, *p <* 0.05). In this context, several retrospective studies have reported that nephrectomy provides benefits for PFS and OS in mRCC by reducing the tumor burden, although there is debate regarding whether this benefit is observed for all patients with mRCC. Thus, the results from two ongoing prospective randomized phase 3 studies may provide definitive data regarding nephrectomy’s efficacy in mRCC that is treated using presurgical or postsurgical TT [22–24]. Nevertheless, the prognostic benefit of nephrectomy during the TT era has generally been positive, as it likely removes a large proportion of the tumor burden and facilitates better responses to TT. In the present study, we found that nephrectomy provided a benefit in 47.6 % of BG patients with favorable-risk features, although this benefit was not significant in the multivariate analysis. In addition, anemia indicated a poor general condition that resembled paraneoplastic syndrome in mRCC, although anemia is known to be a marker for poor inflammatory and immune-related outcomes [8, 9, 25]. Furthermore, the Heng (or IMDC) prognostic model and the MSKCC model include anemia as a poor prognostic factor in their criteria for both PFS and OS [26].

The present study also revealed several significant negative prognostic markers for OS. In the WG, older age (HR: 1.05), decreased hemoglobin (HR: 4.13), lymphocytopenia (HR: 4.76), synchronous metastatic state (HR: 3.52), and a disease-free interval of <1 year were significantly associated with a reduced OS. In the BG, a disease-free interval of <1 year (HR: 0.23), the MSKCC intermediate-risk group (HR: 0.12), and the MSKCC poor-risk group (HR 0.004) were associated with a prolonged OS (all, *p <* 0.05). Previous studies have reported that age is an important prognostic factor for localized RCC, as patients who exhibited late relapse and survival of >5 years beyond expectations were significantly younger, compared to patients who experience early relapse (3 months to 5 years after nephrectomy [27]. Furthermore, patients with RCC who are <40 years old generally have less aggressive tumor features and better survival outcomes [27–29]. Therefore, several studies have suggested that follow-up protocols for younger patients with RCC should be adjusted to include a longer follow-up, as these patients generally experience later relapse [27–29].

Similar to anemia, lymphocytopenia was associated with shortened OS in the present study. In this context, lymphocytes play key roles in tumor suppression, which include inducing cytotoxic cell death and the production of cytokines in cancer cells. Therefore, lymphocytopenia may indicate an impaired antitumor response, and explain the poor prognosis for patients with mRCC [30, 31]. However, the calcium was not significant prognostic factor in any comparisions among BG, WG vs. CG (Tables 3, 4 and 5). The reason for insignificant prognostic role of hypercalcemia like other Heng and MSKCC prognostic models was estimated by the small numbers of hypercalcemia in this study (4.1 %) similar to that of our previously publishing papers (9.4 %) with sunitinib study [32] that the hypercalcemia was not significant either.

In previous studies of various malignancies (including mRCC), the presence of synchronous or metachronous metastasis (based on the time between the diagnoses of the primary and secondary tumor) was a negative prognostic factor for OS. For example, Kwack et al. demonstrated that the time to metastasis and the number of metastases were important prognostic factors for mRCC during the immunotherapy era [29]. Furthermore, the International Metastatic Renal Cell Carcinoma Database Consortium also demonstrated that an increased metastatic tumor burden at the initial therapy was associated with worst OS among all patients with mRCC, and that bone and liver metastases were more frequent in the groups with poor-risk features [26]. Although we did not observe significant differences in the baseline metastatic bone or liver lesions between the three groups, bone and liver metastases were more common in the WG (liver: 26.9 %, bone: 38.6 %), compared to the EG (liver: 12.2 %, bone: 34.0 %) and the BG (liver: 4.5 %, bone: 23.7 %) (all, *p >* 0.05).

This study included several limitations that warrant consideration. First, the retrospective design and small sample size are prone to well-known biases, and larger prospective studies should be performed to validate our findings. Second, we did not perform any histological analyses, and additional analysis of RCC specimens from patients in the BG and WG might have provided histopathological data regarding prognostic biomarkers. Lastly, other existing clinical, political, and economic confounding factors influenced on the prognosis of mRCC during 8-year period of follow-up were not dealt in this study. The improving care system in nutritional, pain, and symptomatic therapeutic fields; introduction of new various curative and palliative strategies such as radiotherapy and metastatectomy, and widening coverage of insurance system on mRCC were the most affecting factors on improvement of prognoses in mRCC, which should be discussed in future studies. Nevertheless, our study identified several factors that were associated with unexpectedly prolonged or shortened survival outcomes after first-line TT treatment, by comparing the BG and WG to the EG. Our findings may provide clinicians with objective markers to identify candidates that are most and least likely to benefit from TTs. Furthermore, our findings may be useful for developing additional prognostic models or helping previous models to potentiate their accuracy of prognostic predictability and therapeutic plans that accurately predict patients’ clinical outcomes in the TT era. For example, the nephrectomy, the presence of synchronous metastasis, age, and lymphocyte level might be also helpful in the MSKCC, Heng model to potentiate its predictability in mRCC treated with first line TT. This study comprised of 46 % patients who had not received nephrectomy, whereas previous Heng criteria comprised of almost all nephrectomized patients that some discrepancies existed when evaluating the non-nephrectomized patients’ prognoses. Therefore, some additionally useful information of non-nephrectomized patients’ prognoses would be obtained in this study.

## Conclusion

The present study identified several significant predictive factors that were associated with unexpectedly prolonged and shortened survival outcomes after first-line TT treatment in patients with mRCC. However, a larger prospective study is needed to validate these factors.

## Abbreviations

BG, the best responsive group; CI, confidence intervals; EG, normal expected responsive group; mRCC, metastatic renal cell carcinoma; MSKCC, Memorial Sloan Kettering Cancer Center; OR, odds ratio; OS, overall survival; PFS, progression-free survival; TT, target therapy; WG, the worst responsive group

## Additional file

Additional file 1:
**Table S1.** Predictive factors for progression-free survival after comparing the expected group and the group with the best response to therapy. **Table S2.** Predictive factors for overall survival after comparing the expected group and the group with the best response to therapy. **Table S3.** Predictive factors for progression-free survival after comparing the expected group and the group with the worst response to therapy. **Table S4.** Predictive factors for overall survival after comparing the expected group and the group with the worst response to therapy. (DOCX 49KB)
