# Supplementary material for: A retrospective study of predictive factors for unexpectedly prolonged or shortened progression-free survival and overall survival among patients with metastatic renal cell carcinoma who received first-line targeted therapy
Source: BMC Cancer. 2016 Aug 2;16:577. doi: 10.1186/s12885-016-2615-4 (PMC4969738; doi:10.1186/s12885-016-2615-4)
Supplement: Additional file 1: — Table S1. Predictive factors for progression-free survival after comparing the expected group and the group with the best response to therapy. Table S2. Predictive factors for overall survival after comparing the expected group and the group with the best response to therapy. Table S3. Predictive factors for progression-free survival after comparing the expected group and the group with the worst response to therapy. Table S4. Predictive factors for overall survival after comparing the expected group and the group with the worst response to therapy. (DOCX 49KB) [file 12885_2016_2615_MOESM1_ESM.docx]

Table S1. Predictive factors for progression-free survival after comparing the expected group and the group with the best response to therapy

|  |  |  |  | **MSKCC risk patients** | | | **Heng risk patients** | | |
| --- | --- | --- | --- | --- | --- | --- | --- | --- | --- |
| **Variables** | **OR** | **P-value** | **95% CI** | **HR** | **P-value** | **95% CI** | **HR** | **P-value** | **95% CI** |
| Age | 1.02 | 0.397 | 0.97-1.07 |  |  |  |  |  |  |
| BMI | 1.20 | 0.085 | 0.98- 1.48 |  |  |  |  |  |  |
| Female gender | 0.27 | 0.224 | 0.03- 2.23 |  |  |  |  |  |  |
| Hypertension | 1.70 | 0.324 | 0.59- 4.85 |  |  |  |  |  |  |
| Diabetes | 0.20 | 0.133 | 0.02- 1.63 |  |  |  |  |  |  |
| MSKCC Intermediate | 0.21 | 0.020 | 0.06- 0.78 |  |  |  |  |  |  |
| Poor | 0.06 | 0.067 | 0.01- 1.23 |  |  |  |  |  |  |
| Heng Intermediate risk group | 0.25 | 0.019 | 0.08- 0.8 |  |  |  | 0.32 | 0.083 | 0.09-1.16 |
| Poor | 0.09 | 0.111 | 0.01- 1.76 |  |  |  | 0.25 | 0.414 | 0.01-7.0 |
| WBC high | 0.84 | 0.836 | 0.16- 4.42 |  |  |  |  |  |  |
| low | 1.12 | 0.925 | 0.11- 11.62 |  |  |  |  |  |  |
| Hemoglobin low | 0.45 | 0.174 | 0.14-1.43 |  |  |  |  |  |  |
| Platelet high | 0.19 | 0.262 | 0.01- 3.48 |  |  |  |  |  |  |
| low | 0.57 | 0.717 | 0.03- 12.37 |  |  |  |  |  |  |
| Lymphocyte high | 0.52 | 0.565 | 0.06- 4.18 |  |  |  |  |  |  |
| Low | 0.19 | 0.119 | 0.02- 56.55 |  |  |  |  |  |  |
| Calcium high | 0.43 | 0.581 | 0.02- 4.83 |  |  |  |  |  |  |
| low | 0.43 | 0.581 | 0.02- 1.54 |  |  |  |  |  |  |
| Albumin low | 0.35 | 0.484 | 0.02- 8.71 |  |  |  |  |  |  |
| Neutrophil (%) high | 0.84 | 0.876 | 0.09- 8.71 |  |  |  |  |  |  |
| low | 2.99 | 0.105 | 0.80- 6.75 |  |  |  |  |  |  |
| DFI < 1yr | 0.22 | 0.006 | 0.07-0.65 |  |  |  |  |  |  |
| Nx | 7.31 | 0.012 | 1.56- 34.33 | 7.15 | 0.016 | 1.43-35.67 | 3.90 | 0.076 | 0.87-17.56 |
| Clinical stage T2 | 0.13 | 0.103 | 0.01- 1.52 |  |  |  |  |  |  |
| T3 | 0.30 | 0.155 | 0.06- 1.58 |  |  |  |  |  |  |
| T4 | 1.00 |  |  |  |  |  |  |  |  |
| TX | 0.67 | 0.646 | 0.12-3.75 |  |  |  |  |  |  |
| N1 | 1.55 | 0.636 | 0.25-9.52 |  |  |  |  |  |  |
| Nx | 1.79 | 0.358 | 0.52- 6.19 |  |  |  |  |  |  |
| synchronous metastasis | 0.36 | 0.064 | 0.12- 1.06 |  |  |  |  |  |  |
| Pathologic stage T2 | 0.09 | 0.139 | 0.01- 2.16 |  |  |  |  |  |  |
| T3 | 0.45 | 0.314 | 0.10- 2.11 |  |  |  |  |  |  |
| T4 | 0.24 | 0.400 | 0.01-6.51 |  |  |  |  |  |  |
| TX | 1.44 | 0.669 | 0.27- 7.79 |  |  |  |  |  |  |
| N1 | 6.00 | 0.165 | 0.48- 75.34 |  |  |  |  |  |  |
| Nx | 1.80 | 0.372 | 0.5- 6.54 |  |  |  |  |  |  |
| Histology: Non-clear | 1.61 | 0.775 | 0.06- 42.03 |  |  |  |  |  |  |
| Papillary | 3.45 | 0.164 | 0.60- 17.7 |  |  |  |  |  |  |
| Sarcomatoid | 2.90 | 0.326 | 0.35- 24.16 |  |  |  |  |  |  |
| primary Treatment: Sorafenib | 0.59 | 0.580 | 0.09- 3.4 |  |  |  |  |  |  |
| Pazopanib | 1.12 | 0.853 | 0.33- 3.83 |  |  |  |  |  |  |
| Temsirolimus | 1.12 | 0.944 | 0.04-29.2 |  |  |  |  |  |  |

Table S2. Predictive factors for overall survival after comparing the expected group and the group with the best response to therapy

|  |  |  |  | **MSKCC risk patients** | | | **Heng risk patients** | | |
| --- | --- | --- | --- | --- | --- | --- | --- | --- | --- |
| **Variables** | **OR** | **P-value** | **95% CI** | **HR** | **P-value** | **95% CI** | **HR** | **P-value** | **95% CI** |
| Age | 0.99 | 0.814 | 0.95-1.04 |  |  |  |  |  |  |
| BMI | 1.10 | 0.349 | 0.90 -1.35 |  |  |  |  |  |  |
| Female gender | 1.46 | 0.554 | 0.42- 5.13 |  |  |  |  |  |  |
| Hypertension | 0.97 | 0.956 | 0.38- 2.49 |  |  |  |  |  |  |
| Diabetes | 1.17 | 0.786 | 0.37- 3.67 |  |  |  |  |  |  |
| MSKCC Intermediate | 0.12 | 0.001 | 0.03- 0.44 | 0.12 | 0.003 | 0.03-0.49 |  |  |  |
| Poor | 0.04 | 0.040 | 0.01-0.86 | 0.04 | 0.041 | 0.01-0.87 |  |  |  |
| Heng Intermediate risk group | 0.29 | 0.027 | 0.10- 0.87 |  |  |  |  |  |  |
| Poor | 0.12 | 0.175 | 0.01- 2.58 |  |  |  |  |  |  |
| WBC high | 0.98 | 0.978 | 0.16- 5.79 |  |  |  |  |  |  |
| low | 0.98 | 0.984 | 0.08- 11.41 |  |  |  |  |  |  |
| Hemoglobin low | 0.88 | 0.809 | 0.31-2.51 |  |  |  |  |  |  |
| Platelet high | 0.19 | 0.268 | 0.01- 3.63 |  |  |  |  |  |  |
| low | 0.34 | 0.488 | 0.02- 7.31 |  |  |  |  |  |  |
| Lymphocyte high | 2.10 | 0.474 | 0.28- 16.01 |  |  |  |  |  |  |
| Low | 2.10 | 0.606 | 0.12-35.32 |  |  |  |  |  |  |
| Calcium high | 0.63 | 0.780 | 0.02- 7.2 |  |  |  |  |  |  |
| low | 1.13 | 0.906 | 0.14- 4.67 |  |  |  |  |  |  |
| Albumin low | 0.37 | 0.527 | 0.02- 16.1 |  |  |  |  |  |  |
| Neutrophil (%) high | 6.82 | 0.247 | 0.26- 9.14 |  |  |  |  |  |  |
| low | 1.74 | 0.356 | 0.54- 8.0 |  |  |  |  |  |  |
| DFI < 1yr | 0.22 | 0.003 | 0.08- 176.29 |  |  |  | 0.23 | 0.013 | 0.07-0.73 |
| Nx | 7.81 | 0.009 | 1.67-5.6 |  |  |  |  |  |  |
| Clinical stage T2 | 2.92 | 0.211 | 0.54- 0.6 |  |  |  |  |  |  |
| T3 | 1.23 | 0.784 | 0.28- 36.62 |  |  |  |  |  |  |
| T4 | 0.24 | 0.377 | 0.01- 15.67 |  |  |  |  |  |  |
| TX | 1.24 | 0.798 | 0.24- 5.52 |  |  |  |  |  |  |
| N1 | 0.45 | 0.350 | 0.08- 5.76 |  |  |  |  |  |  |
| Nx | 0.85 | 0.790 | 0.26-6.44 |  |  |  |  |  |  |
| synchronous metastasis | 0.29 | 0.015 | 0.10- 2.42 |  |  |  |  |  |  |
| Pathologic stage T2 | 3.57 | 0.148 | 0.64- 2.76 |  |  |  |  |  |  |
| T3 | 1.09 | 0.904 | 0.26- 0.78 |  |  |  |  |  |  |
| T4 | 0.33 | 0.508 | 0.01-20.04 |  |  |  |  |  |  |
| TX | 1.41 | 0.679 | 0.28- 4.55 |  |  |  |  |  |  |
| N1 | 1.44 | 0.730 | 0.18- 8.63 |  |  |  |  |  |  |
| Nx | 1.44 | 0.562 | 0.42- 7.19 |  |  |  |  |  |  |
| Histology: Non-clear | 0.85 | 0.921 | 0.03-4.9 |  |  |  |  |  |  |
| Papillary | 3.56 | 0.147 | 0.64-19.77 |  |  |  |  |  |  |
| Sarcomatoid | 4.24 | 0.177 | 0.52- 34.55 |  |  |  |  |  |  |
| primary Treatment: Sorafenib | 0.56 | 0.501 | 0.10-3.02 |  |  |  |  |  |  |
| Pazopanib | 0.56 | 0.421 | 0.14- 2.29 |  |  |  |  |  |  |

Table S3. Predictive factors for progression-free survival after comparing the expected group and the group with the worst response to therapy

|  |  |  |  | **MSKCC risk patients** | | | **Heng risk patients** | | |
| --- | --- | --- | --- | --- | --- | --- | --- | --- | --- |
| **Variables** | **OR** | **P-value** | **95% CI** | **HR** | **P-value** | **95% CI** | **HR** | **P-value** | **95% CI** |
| Age | 1.00 | 0.800 | 0.97-1.04 |  |  |  |  |  |  |
| BMI | 1.02 | 0.777 | 0.89- 1.17 |  |  |  |  |  |  |
| Female gender | 1.63 | 0.258 | 0.70- 1.26 |  |  |  |  |  |  |
| Hypertension | 1.08 | 0.835 | 0.53- 2.18 |  |  |  |  |  |  |
| Diabetes | 1.21 | 0.646 | 0.54- 2.72 |  |  |  |  |  |  |
| MSKCC Intermediate | 1.19 | 0.821 | 0.26-5.37 |  |  |  |  |  |  |
| Poor | 4.29 | 0.089 | 0.8- 22.92 |  |  |  |  |  |  |
| Heng Intermediate risk group | 1.63 | 0.417 | 0.5-5.29 |  |  |  |  |  |  |
| Poor | 3.90 | 0.068 | 0.91- 16.79 |  |  |  |  |  |  |
| WBC high | 2.04 | 0.135 | 0.8 5.18 |  |  |  |  |  |  |
| low | 0.16 | 0.229 | 0.01- 3.18 |  |  |  |  |  |  |
| Hemoglobin low | 3.29 | 0.003 | 1.49- 7.27 | 3.25 | 0.006 | 1.41-7.52 | 2.87 | 0.014 | 1.23-6.66 |
| Platelet high | 1.75 | 0.288 | 0.62- 4.91 |  |  |  |  |  |  |
| low | 1.11 | 0.916 | 0.15- 8.24 |  |  |  |  |  |  |
| Lymphocyte high | 0.56 | 0.503 | 0.1-3.08 |  |  |  | 0.26 | 0.242 | 0.03-2.47 |
| Low | 2.69 | 0.016 | 1.2- 6.02 |  |  |  | 2.05 | 0.098 | 0.88-4.78 |
| Calcium high | 1.21 | 0.821 | 0.23- 6.3 |  |  |  |  |  |  |
| low | 4.43 | 0.029 | 1.16- 16.92 |  |  |  |  |  |  |
| Albumin low | 2.20 | 0.219 | 0.62- 7.78 |  |  |  |  |  |  |
| Neutrophil (%) high | 2.35 | 0.140 | 0.76- 7.33 |  |  |  |  |  |  |
| low | 0.46 | 0.280 | 0.11-1.89 |  |  |  |  |  |  |
| DFI < 1yr | 1.26 | 0.588 | 0.55- 2.86 |  |  |  |  |  |  |
| Nx | 0.78 | 0.474 | 0.39- 1.55 |  |  |  |  |  |  |
| Clinical stage T2 | 0.50 | 0.410 | 0.1- 2.6 |  |  |  |  |  |  |
| T3 | 0.47 | 0.299 | 0.11- 1.97 |  |  |  |  |  |  |
| T4 | 1.33 | 0.746 | 0.23- 7.63 |  |  |  |  |  |  |
| TX | 0.44 | 0.330 | 0.09- 2.28 |  |  |  |  |  |  |
| N1 | 4.23 | 0.014 | 1.34- 13.35 |  |  |  |  |  |  |
| Nx | 0.76 | 0.613 | 0.26- 2.21 |  |  |  |  |  |  |
| synchronous metastasis | 1.28 | 0.536 | 0.59- 2.78 |  |  |  |  |  |  |
| Pathologic stage T2 | 1.00 | 1.000 | 0.19- 5.36 |  |  |  |  |  |  |
| T3 | 0.65 | 0.557 | 0.15-2.77 |  |  |  |  |  |  |
| T4 | 1.50 | 0.715 | 0.17- 13.23 |  |  |  |  |  |  |
| TX | 0.60 | 0.597 | 0.09- 3.99 |  |  |  |  |  |  |
| N1 | 6.32 | 0.105 | 0.68-58.72 |  |  |  |  |  |  |
| Nx | 0.51 | 0.306 | 0.14- 1.87 |  |  |  |  |  |  |
| Histology: Non-clear | 2.44 | 0.471 | 0.21- 27.84 |  |  |  |  |  |  |
| Papillary | 2.44 | 0.224 | 0.58- 10.33 |  |  |  |  |  |  |
| Sarcomatoid | 1.22 | 0.844 | 0.17- 11.45 |  |  |  |  |  |  |
| primary Treatment: Sorafenib | 1.05 | 0.933 | 0.36-3.04 |  |  |  |  |  |  |
| Pazopanib | 0.64 | 0.376 | 0.24-1.71 |  |  |  |  |  |  |
| Temsirolimus | 2.09 | 0.552 | 0.18-23.93 |  |  |  |  |  |  |

Table S4. Predictive factors for overall survival after comparing the expected group and the group with the worst response to therapy

|  |  |  |  | **MSKCC risk patients** | | | **Heng risk patients** | | |
| --- | --- | --- | --- | --- | --- | --- | --- | --- | --- |
| **Variables** | **OR** | **P-value** | **95% CI** | **HR** | **P-value** | **95% CI** | **HR** | **P-value** | **95% CI** |
| Age | 1.03 | 0.099 | 0.99-1.06 | 1.05 | 0.045 | 1.01-1.10 |  |  |  |
| BMI | 0.90 | 0.161 | 0.78- 1.04 |  |  |  |  |  |  |
| Female gender | 2.19 | 0.113 | 0.83-5.75 |  |  |  |  |  |  |
| Hypertension | 0.99 | 0.972 | 0.47-2.05 |  |  |  |  |  |  |
| Diabetes | 1.29 | 0.580 | 0.53-3.13 |  |  |  |  |  |  |
| MSKCC Intermediate | 10.30 | 0.122 | 0.54- 198.3 |  |  |  |  |  |  |
| Poor | 33.55 | 0.025 | 1.56- 722.3 |  |  |  |  |  |  |
| Heng Intermediate risk group | 6.47 | 0.022 | 1.31- 31.95 |  |  |  |  |  |  |
| Poor | 24.00 | 0.002 | 3.36- 171.54 |  |  |  |  |  |  |
| WBC high | 4.21 | 0.015 | 1.32- 13.45 |  |  |  |  |  |  |
| low | 0.44 | 0.513 | 0.04- 5.08 |  |  |  |  |  |  |
| Hemoglobin low | 6.46 | 0.000 | 2.74- 15.22 | 4.13 | 0.008 | 1.44-11.8 | 4.61 | 0.003 | 1.68-12.66 |
| Platelet high | 2.84 | 0.085 | 0.87- 9.34 |  |  |  |  |  |  |
| low | 0.81 | 0.839 | 0.11- 6.03 |  |  |  |  |  |  |
| Lymphocyte high | 0.36 | 0.368 | 0.04- 15.22 | 0.22 | 0.215 | 0.02-2.42 | 0.18 | 0.160 | 0.02-1.95 |
| Low | 13.16 | 0.000 | 4.18- 9.34 | 4.76 | 0.022 | 1.25-18.17 | 5.26 | 0.012 | 1.44-19.14 |
| Calcium high | 4.37 | 0.186 | 0.49- 6.03 |  |  |  |  |  |  |
| low | 4.81 | 0.049 | 1.01- 3.37 |  |  |  |  |  |  |
| Albumin low | 3.98 | 0.085 | 0.83- 41.45 |  |  |  |  |  |  |
| Neutrophil (%) high | 26.05 | 0.025 | 1.51- 38.96 |  |  |  |  |  |  |
| low | 0.14 | 0.031 | 0.02- 22.96 |  |  |  |  |  |  |
| DFI < 1yr | 3.81 | 0.007 | 1.43- 10.15 | 4.80 | 0.036 | 1.10-20.9 |  |  |  |
| Nx | 0.31 | 0.002 | 0.14-0.65 |  |  |  |  |  |  |
| Clinical stage T2 | 1.05 | 0.960 | 0.16-6.92 |  |  |  |  |  |  |
| T3 | 1.64 | 0.493 | 0.40- 6.76 |  |  |  |  |  |  |
| T4 | 3.50 | 0.153 | 0.63- 19.5 |  |  |  |  |  |  |
| TX | 1.36 | 0.701 | 0.28- 6.58 |  |  |  |  |  |  |
| N1 | 2.32 | 0.130 | 0.78- 6.91 |  |  |  |  |  |  |
| Nx | 1.02 | 0.967 | 0.35-2.95 |  |  |  |  |  |  |
| synchronous metastasis | 4.68 | 0.001 | 1.85- 11.84 | 3.52 | 0.039 | 1.07-11.61 | 3.17 | 0.057 | 0.97-10.41 |
| Pathologic stage T2 | 1.56 | 0.699 | 0.17- 14.65 |  |  |  |  |  |  |
| T3 | 1.83 | 0.448 | 0.38- 8.78 |  |  |  |  |  |  |
| T4 | 3.50 | 0.274 | 0.37- 32.97 |  |  |  |  |  |  |
| TX | 1.17 | 0.876 | 0.17- 8.09 |  |  |  |  |  |  |
| N1 | 3.83 | 0.151 | 0.61- 24.02 |  |  |  |  |  |  |
| Nx | 1.64 | 0.452 | 0.45- 6.0 |  |  |  |  |  |  |
| Histology: Non-clear | 1.76 | 0.649 | 0.15- 20.08 |  |  |  |  |  |  |
| Papillary | 2.64 | 0.249 | 0.51- 13.76 |  |  |  |  |  |  |
| Sarcomatoid | 1.76 | 0.649 | 0.15- 26.31 |  |  |  |  |  |  |
| primary Treatment: Sorafenib | 1.29 | 0.647 | 0.43-3.84 |  |  |  |  |  |  |
| Pazopanib | 1.25 | 0.637 | 0.49-3.21 |  |  |  |  |  |  |
| Temsirolimus | 6.18 | 0.234 | 0.31- 123.5 |  |  |  |  |  |  |
